# An electron microscopic study of the archaeal feast/famine regulatory protein: 7. Single particles formed by pot0434017

**Published:** 2004-10-01

**Authors:** Sanae A. Ishijima, Lester Clowney, Masashi Suzuki

**Affiliations:** *) National Institute of Advanced Industrial Science and Technology (AIST), AIST Tsukuba Center 6-10, Higashi 1-1-1, Tsukuba, Ibaraki 305-8566, Japan; **) Japan Science and Technology Agency (JST), Core Research for Evolutionary Science and Technology (CREST), Honmachi 4-1-18, Kawaguchi Center Building, Kawaguchi, Saitama 332-0012, Japan

**Keywords:** Cryo-electron microscopy, cryo-electron staining, image processing, single particle analysis, 3D reconstruction, transcription factor

## Abstract

Particles formed by a feast/famine regulatory protein (FFRP), pot0434017 (FL11), in solution in the absence of DNA were analyzed using electron microscopy (EM). By applying conventional (i.e. dry) EM to the protein negatively stained with uranyl acetate, top views of tetrameric assemblies of dimers were obtained, where four pairs each of N-domains were extending from C-domains assembled around the centers. In cryo-EM images of the protein embedded in 3D amorphous ice, sets of four densities were arranged around ellipsoids having similar lengths for their long axes but of different lengths for their short axes. These images were interpreted as projections with different tilts of four pairs of N-domains arranged inside flat assemblies: the positively charged N-domains only were stained with ammonium molybdate, but the negatively charged C-domains were unstained and thus unobservable. Using seventeen such cryo-images, in combination with a crystal structure equivalent to an assembly of C-domains, a disk-like 3D structure was reconstructed.

## Introduction

While studying the structure and function of a group of bacterial transcription factors, the feast/famine regulatory proteins (FFRPs),1)–14) we have crystallized an FFRP, pot0434017 (FL11) from the hyper-thermophilic archaeon *Pyrococcus* sp. OT3, into cylinder-like assemblies, and, by using X-ray diffraction, we have determined the 3D structure of these cylinders, where six dimers each formed a helical turn.3)

In solution, a cylinder is not formed by the same protein2): an observation obtained using cryo-electron microscopy with the cryo-staining method. Instead, in the absence of DNA molecules, FL11 predominantly forms dimers but larger particles are also formed.2) The diameters of these particles were estimated to be ~130 Å by analyzing the electron micrographs using a Fourier method.5) When DNA molecules were added, dimers disappeared and particles became predominant.2)

Compared with the sizes and morphology of crystal assemblies of the same and other FFRPs, it is likely that the particles of FL11 are disk-like assemblies of octamers (i.e. four pairs of dimers), either binding or not binding DNA2): here referred to as tetrameric assemblies. In fact, the tetrameric assembly form is most common among FFRPs in crystal as well as in solution: *E. coli* LRP,15) LrpA,16) and pot1216151 (DM1).13),14)

In short, FL11 has multiple assembly forms, and the aim of this paper is to further characterize particles (i.e. possible tetrameric assemblies) formed in solution in the absence of DNA, partly by re-analyzing the cryo-electron micrographs.

## Electron microscopy

The protein pot0434017 (FL11) was expressed and purified.2) For conventional (i.e. non-cryo) electron microscopy, 50 mM Na-phosphate buffer (pH = 8.0) containing 10 mg/ml FL11 and 300 mM NaCl was mixed with the same volume of 50 mM Na-phosphate buffer (pH = 8.0) containing 2 M ammonium sulfate. A droplet, 2 μl, of the mixture was placed onto a microgrid (300 mesh and collodion-coated, Electron Microscopy Sciences Co.) coated with carbon particles (250 Å) and evaporated in vacuum. The solvent was removed immediately by using a piece of filter paper. The same volume, 2 μl, of 2% uranyl acetate solution (pH = 4.2) was added. After 30 seconds, the solvent was removed by using a piece of filter paper. While operating the same electron microscope, defocusing by 0.76 μm was used, the first CTF minimum corresponded to 17.7 Å. The magnification on the CCD plane was 158 k, each pixel covering 1.54 Å × 1.54 Å.

The cryo-electron micrographs analyzed are the same as those used in our previous analysis2),5) (see reference 17 and other reviews 18–21 for understanding cryo-electron microscopy and reference 22, in particular, for cryo-electron staining). A droplet, 4 μl, of each protein solution, 34 μg/ml in 10 mM Tris-HCl buffer (pH = 7.5) containing 133 mM KCl, was placed on a holey copper grid (carbon-coated and 300 mesh, Electron Microscopy Sciences Co.). In order to enhance the contrast of images, the protein was stained using 16% ammonium molybdate, by the method of Adrian *et al*.22) The grid was quickly frozen in liquid ethane into an amorphous ice state, using a freezing apparatus (EM CPC, Leica). The grid was maintained at a near liquid nitrogen temperature using a holder (CT3500, Oxford) in order to minimize damage caused by dehydration and electron irradiation, while an electron microscope (Tecnai F20, FEI) was operated at 200 keV. Electrons at a low density (~5 electrons/Å^2^) were irradiated, and electrons (200 ± 10 keV) that had lost no energy upon interaction with the specimen were selectively focused using an energy filter (GIF200, Gatan), thereby minimizing chromatic aberration. Defocusing by ~3 μm was used in order to enhance the phase contrast: the first CTF minima of the four electron micrographs taken were 27.7–34.8 Å. A CCD camera (794IF, Gatan, 1,024 pixels × ~1,024 pixels) and Digital Micrograph software (Gatan) were used for recording the images. The magnification on the CCD plane was 158 k, each pixel covering 1.54 Å × 1.54 Å.

All the electron micrographs, conventional or cryo, were processed by low pass filtration23) to remove noise beyond the first CTF minima, using the proc2d program in the eman package.24)

## Tetrameric assemblies of FL11

**Fig. 1:**
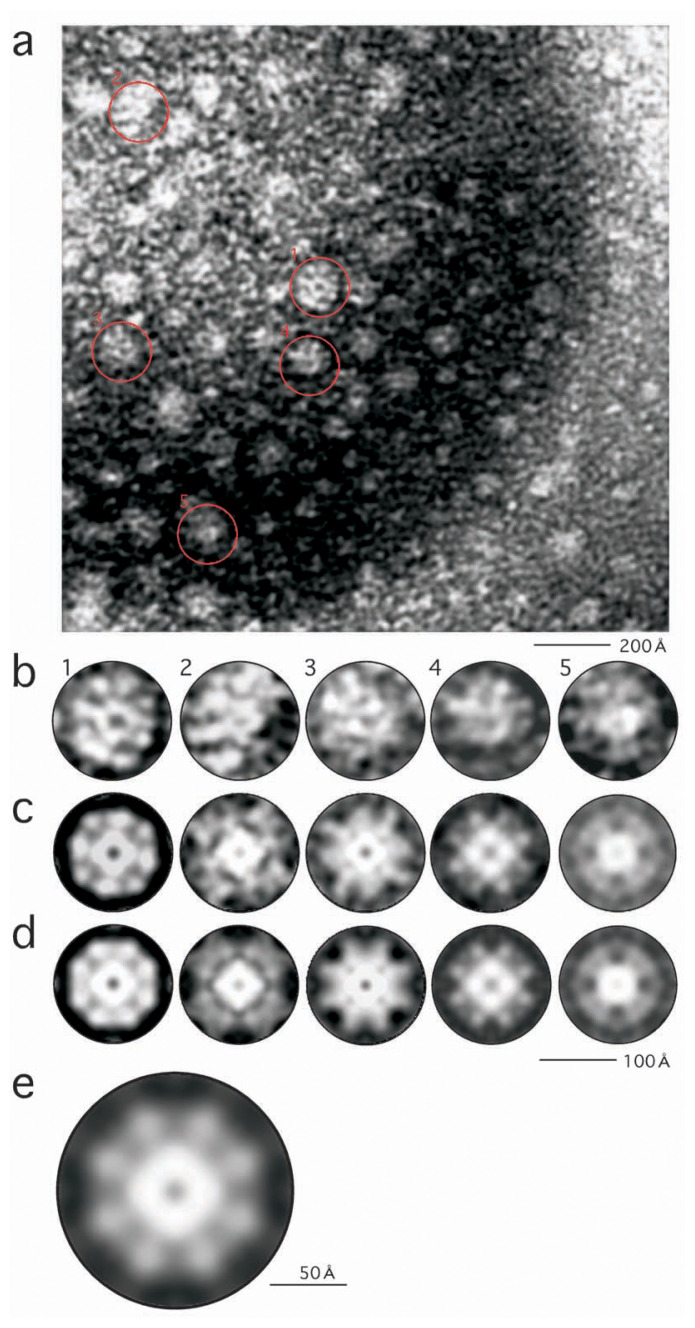
Conventional EM images of FL11 negatively stained using uranyl acetate. (a) The whole view of an electron micrograph. Particles enlarged in (b) are circled. (b) Five particles (1–5) selected from (a). (c) Particles shown in (b) symmetrized by imposing four-fold symmetries around their centers. (d) Particles shown in (c) averaged with their mirror images, producing 4·2·2 symmetries. (e) An average of the five images in (d).

Using a conventional negative staining method, where protein molecules were dried, surrounded by electron-dyes, an electron micrograph showing particles was obtained (Fig. 1a). Here the solution used for staining was acidic (pH = 4.2), and the two domains of FL11, N-terminal (pI = 10.0) and C-terminal (pI = 5.6), both were positively charged and thus were repulsive to UO_2_^2+^.

**Fig. 2:**
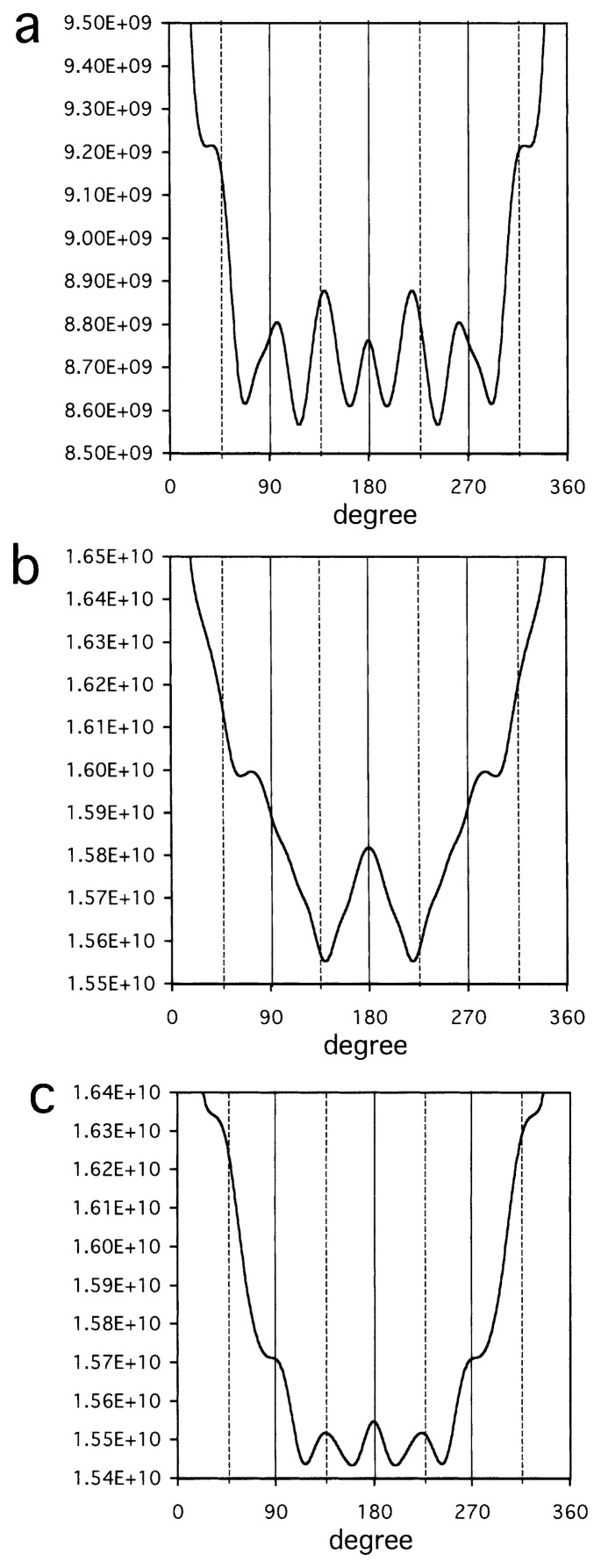
Autocorrelation functions calculated for images 1, 5 in Fig. 1b (a and b, respectively) and the average of those calculated for images 1–5 in Fig. 1b (c). The presence of peaks at the angles 90°, 180°, 270° (—), showing four-fold symmetries, and the presence or absence of peaks at the angles 45°, 135°, 225° and 315° (----), showing or not showing eight-fold symmetries, are highlighted.

These particles share some characteristics (Fig. 1b): i.e. each particle has a substructure circling around a hole at the center, from which a set of globular substructures protruded. When the autocorrelation function was calculated for each particle (Fig. 2a, b), peaks were found at around 90°, 180°, 270°, showing the presence of a four-fold axis around the center. When complete fourfold symmetries were imposed using the eman package, images (Fig. 1c) showed inner circles (or diamonds) connected to four pairs each of globules outside. Additional peaks were present at 45°, 135°, 225°, 315° in the autocorrelation functions with different intensities (Fig. 2), reflecting degrees of separation between two globules in pairs: compare, e.g. Fig. 1c1 with Fig. 1c5.

The five images four-fold symmetrized were overlaid onto each other and averaged, thereby enhancing the shared characteristics (Fig. 1e). Here no characteristic of the images hints at whether each particle was projected from its “top” side or “bottom”: the focus of an electron microscope is deep, and thus what is observed is a projection of the object. In order to consider the two possibilities, each image was averaged with its mirror image (Fig. 1d) prior to the averaging of the five.

More generally, correlation of the four pairs of globules in a particle to the other four in another has ambiguities: pairs 1,2,3,4 in particle A should correspond to pairs 1,2,3,4, respectively, in particle B or 2,3,4,1 or even to 4,3,2,1. In order to consider all of these possibilities, averaging was necessary, effectively by imposing a four-fold symmetry in order to deal with the “1,2,3,4 or 2,3,4,1” type of ambiguities, and another two-fold symmetry around an axis connecting two pairs across the four-fold symmetry in order to deal with the “1,2,3,4 or 4,3,2,1” type of ambiguities.

## Positioning N-domains outside and C-domains inside

In general, when an object is dried by conventional EM, it tends to orient by keeping a particular geometry relative to the grid. Thus cryo-electron micrographs taken earlier2),5) were re-analyzed, where assemblies of FL11 were expected to be more randomly oriented, having been quickly frozen in solution and kept in amorphous ice.

The particular protocol used here22) for cryo-electron microscopy was originally designed for cryo-negative staining. However, the N-domain of FL11 is extremely basic (pI = 10.0), and at a pH of 7.4 in the protein-dye mixture, ammonium molybdate forming anions such as Mo_7_O_24_^6−^ or HMo_7_O_24_^5−^ would preferentially bind to the basic amino acid residues in the N-domain. This absorption of the dye by N-domains would decrease the background density, leaving the C-domain (pI = 5.6) unstained. It should be possible to distinguish images of the two domains recorded by conventional EM in comparison with images recorded by cryo-EM.

**Fig. 3:**
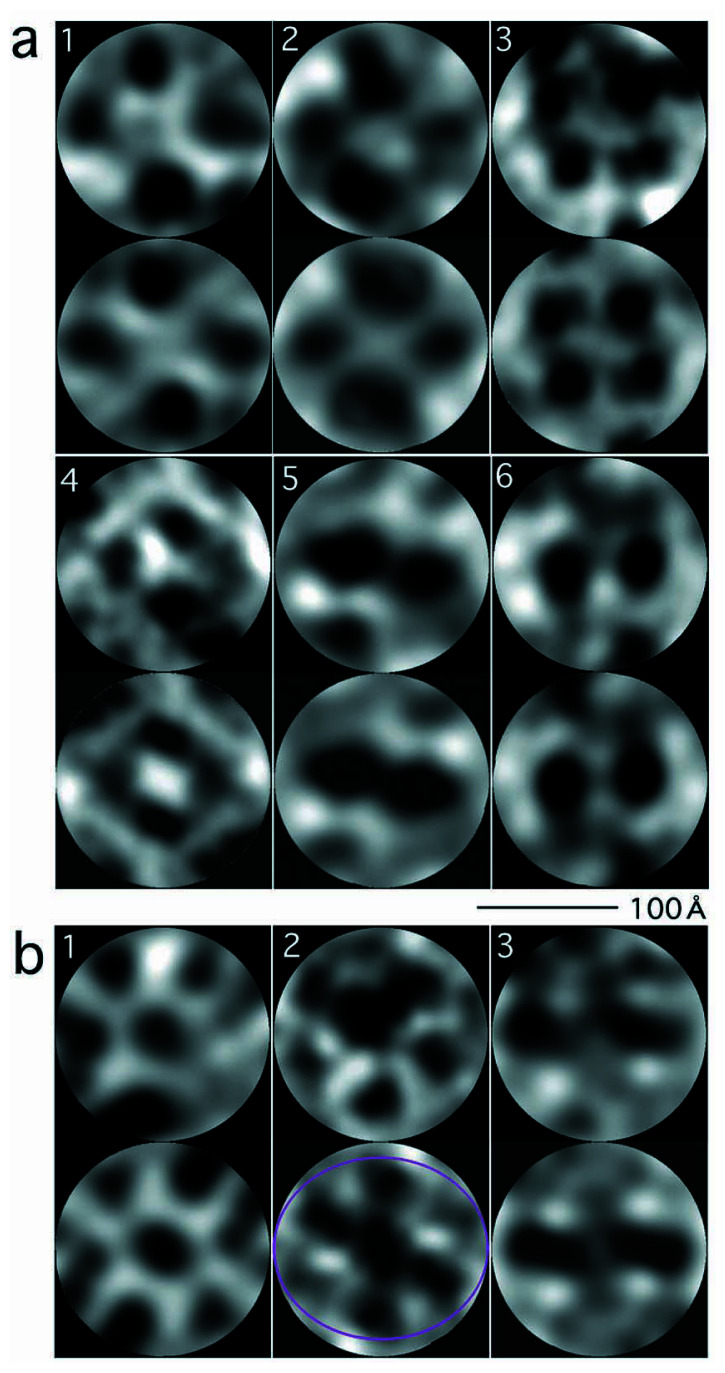
A selection of images before (upper) and after (lower) imposing two-fold symmetries. Those interpreted as projections of smaller (a) and larger (b) assemblies. In (a) 1–6 correspond to images T2, T3, T7, T9, T15 and T17, respectively, in Table I. In (b) 1–3 correspond to images T101–T103, respectively in Table II. To image 2 in (b) lower, an ellipsoid is overlaid, showing a possible ellipsoidal trace of the original disk projected (Fig. 5a). The scale 100 Å is shown.

In the cryo-electron micrographs sets of four densities were identified (Fig. 3a). Images showing two densities only can be interpreted as those where four densities are overlaid, creating two densities only. Inside some of these sets the four densities were arranged around circles of diameters ~100 Å (Fig. 3a1, 2), similar to the diameters for arranging the four pairs of globules in the conventional EM images (Fig. 1e). In some others (Fig. 3a3, 4), as is discussed in the next section, it was possible to draw ellipsoids by connecting the four densities, with the long axes having the same length close to 100 Å (Table I).

Based on the above observations, it seems reasonable to conclude that each of the four densities in each set corresponds to a pair of globules observed by conventional EM. If each pair of globules corresponds to a pair of N-domains, the unit of the four-fold symmetry must be a dimer of FL11. Such pairs of N-domains were not separated from each other by cryo-EM, because of the lower resolution. Also electric charges of these domains are neutralized by the dye used. The process of imposing the 4·2·2 symmetry was not only needed because of the lack of information, but also it is reasonable and necessary in order to obtain some further details of the unit, i.e. a dimer. It is still possible to form a dimer without imposing a two-fold symmetry. However, it is unlikely that such an asymmetric dimer would function uniquely as an independent unit, since, by using similar interactions trimers, tetramers etc. would be formed.

## Tetramerization of FL11 dimers into a disk

It is known that two other FFRPs, when crystallized, form tetramers, where each dimer contacts two other dimers on both sides, the four closing into a disk.14),16) Images such as Fig. 1e can be interpreted as “close to top” views of such a disk-like structure, while images such as Fig. 3a1 (T2 in Table I) and Fig. 3a2 (T3 in Table I) can be interpreted as N-domains only in top views. Images such as Fig. 3a5 (T15 in Table I) and Fig. 3a6 (T17 in Table I), having two densities only, can be interpreted as side views, and images such as Fig. 3a3 (T7 in Table I) and Fig. 3a4 (T9 in Table I) as tilted projections.

**Fig. 4:**
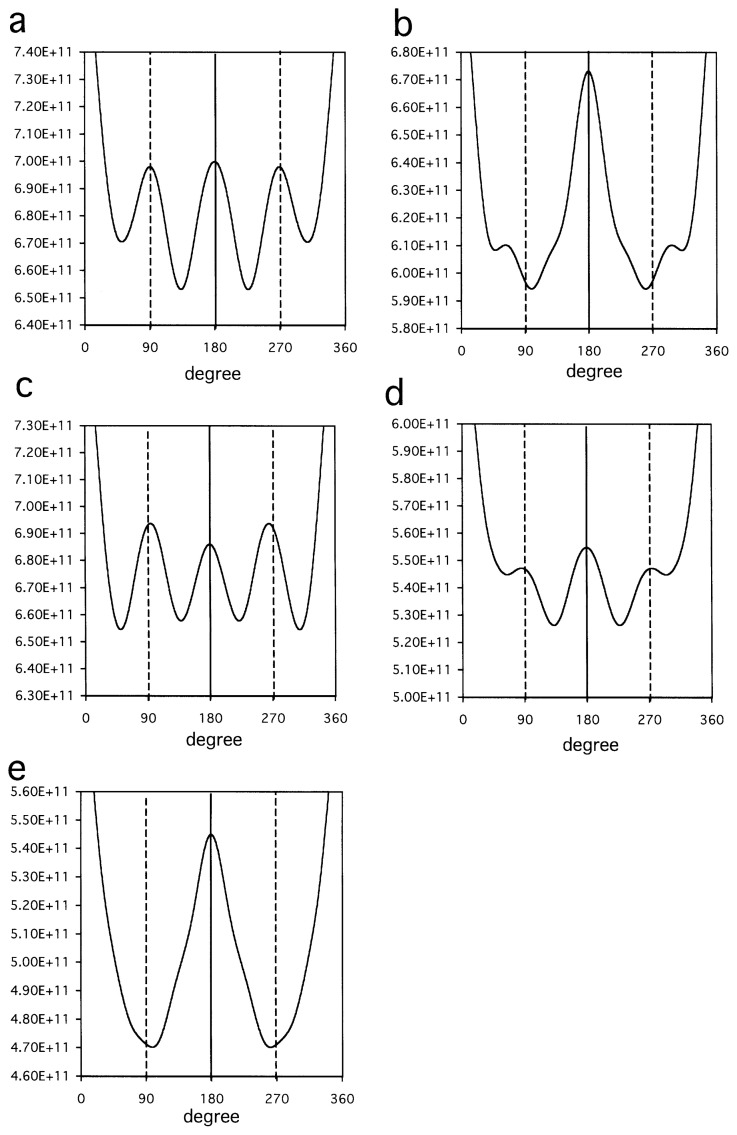
Autocorrelation functions calculated for EM images. Those calculated using images T1 (a) and T15 (b), and averages of those calculated using “close to top” views T1–T5 (c), tilted views T6–T13 (d), and “close to side” views T14–T17 (e), respectively. The presence of peaks at the angle 180° (—), showing two-fold symmetries, and the presence or absence of peaks at the angles 90° and 270° (----), showing or not showing of four-fold symmetries, are highlighted.

**Fig. 5:**
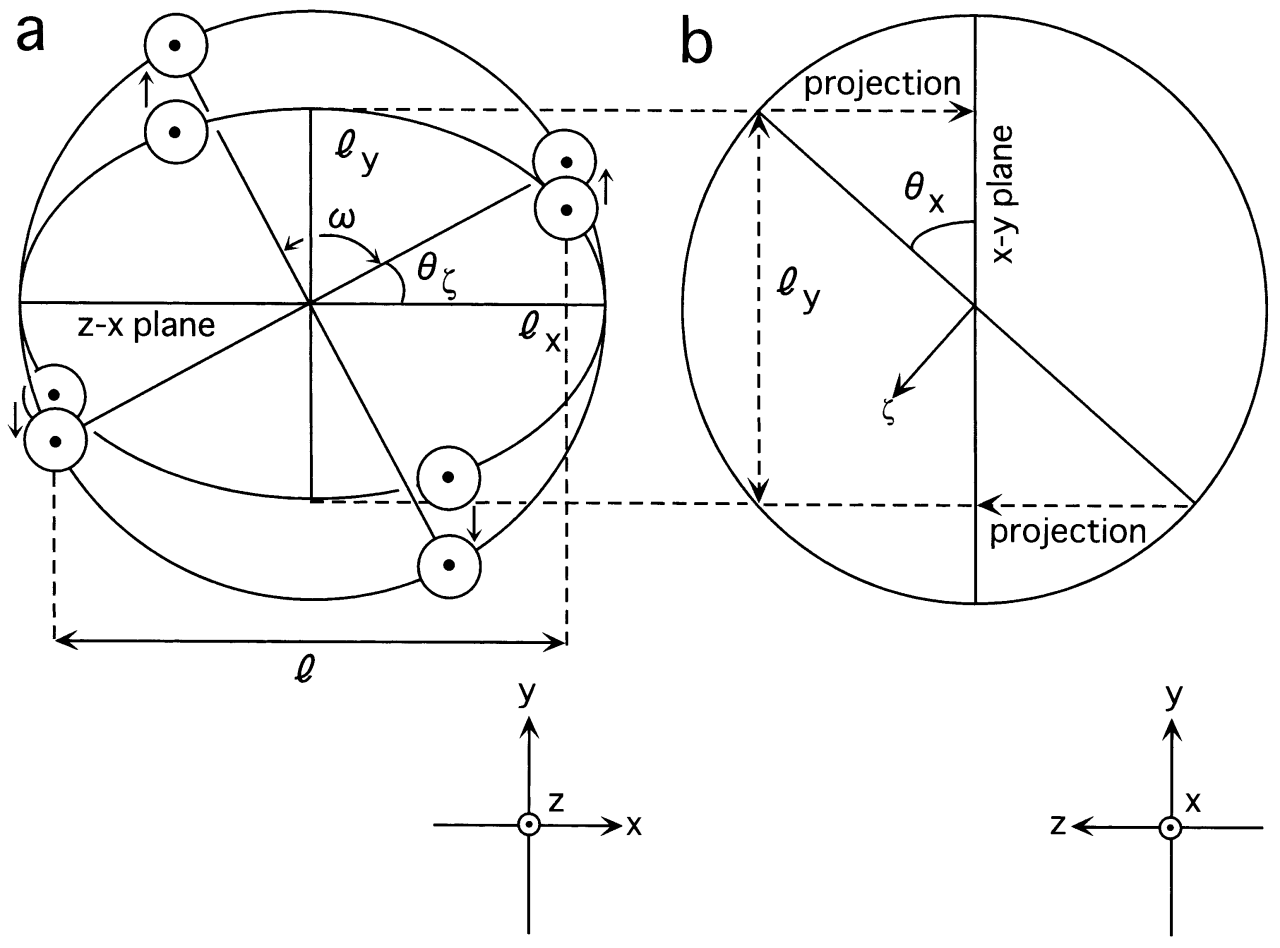
The geometric frame used for describing rotation/tilting of a disk in 3D. Two views (a, b) are shown. For each projection (i.e. the ellipsoidal EM image), the x-y-z axes were defined so that the longer diameter *l*x of the ellipsoid was extending along the chosen x axis, and the shorter diameter *l*y along the y axis. Thus *l*x was the same as the diameter of the disk, and *θ* y angle, defining rotation around the y axis, became 0°. By the ratio of *l*y to *l*x, the tilt angle *θ* x was determined. In order to determine *θ_ζ_* angle, four domains positioned around the ellipsoid were projected back to a circle of the diameter *l*x (a, arrows), so that the *ω* angle, the angle, measured between the two lines connecting the pairs of densities now positioned around the circle (i.e. non-ellipsoidal), should become closest to 90°. *l* is the length of the disk-like density, when projected along the x axis.

In order to confirm the disk-like arrangement, seventeen cryo-images were chosen (Fig. 3a), which have clear autocorrelation at 180° (Fig. 4): any object which has an even-numbered symmetry will have a two-fold symmetry, when projected onto a 2D plane regardless of any tilting. After imposing two-fold symmetries to these images using the eman package (Fig. 3a lower panels in the subfigures), by using a frame (Fig. 5), orientations of the particles were determined (Table I) by assuming the same diameter. This process resulted in very small deviations from what were assumed for perfect four-fold symmetries, in terms of parameter Δ*ω* (see next paragraph), suggesting that the assemblies are indeed disk-like.

In Fig. 3a, images are rotated, so that the chosen y axis runs vertically: *θ* y = 0. The long axis *l*x of an ellipsoid coincides with the diameter of the disk-like assembly. While, the ratio of *l*y (the shorter axis of the ellipsoid) to *l*x determines the *θ* x tilt around the x axis. In addition to *θ* x and *θ_ζ_* (i.e. the rotation around the tilted z axis, namely *ζ* ), *ω* was introduced to define the angle between two lines connecting pairs of densities across the center, when projected back to a disk (Fig. 5a). Ideally this angle should be 90°. The difference between the observed and ideal values of *ω*, Δ*ω*, was always kept very small, 0.0–2.2° (Table I), at the same time when the same *l*x value was assumed, suggesting that the assembly is indeed disk-like. When *θ* x was 90° (i.e. images T16, T17), *θ_ζ_* was determined by measuring the *l* to *l*x ratio, where *l* was the length of the image projected along the x axis (Fig. 5a).

## Three dimensional reconstruction

**Fig. 6:**
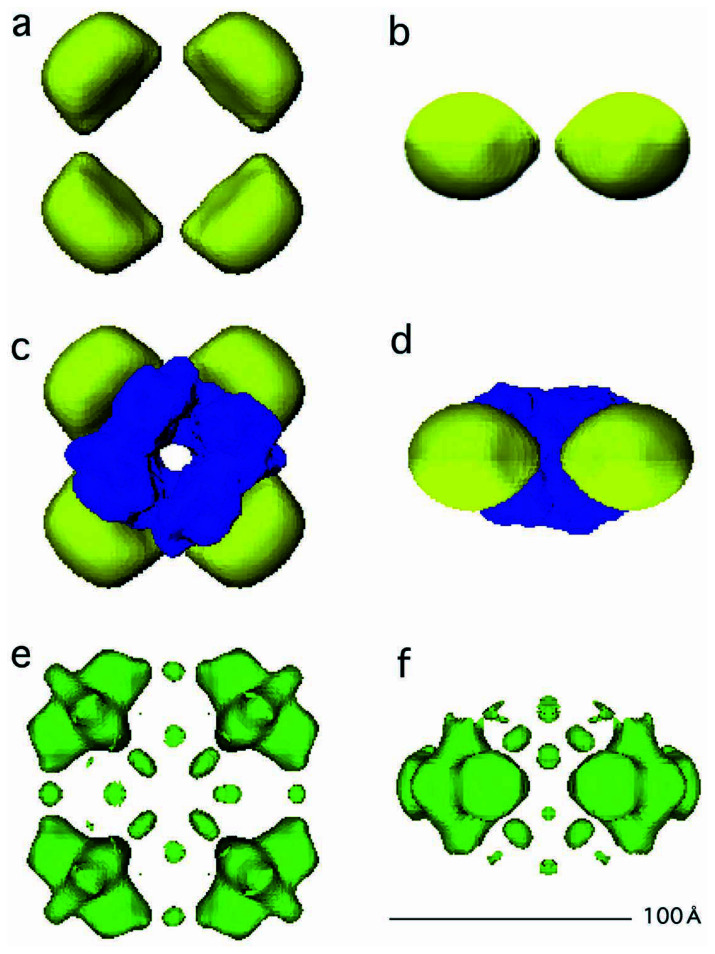
Reconstruction of the 3D structure of a tetrameric assembly of FL11. Top (a) and side (b) views of a surface representation, made using the “open inventor” program,25) of a reconstruction using cryo-EM images. The same top (c) and side (d) views of FL11 (yellow) combined with a Van der Waals model of the crystal structure of DM1 (blue).13),14) Top (e) and side (f) views of a model reconstructed without assuming an Euler-angle. An initial model was made by assuming a four-fold symmetry, using a program, StartAny, and the same seventeen EM images two-fold symmetrized but not low pass-filtered. The model was averaged with its mirror structure, yielding a 4·2·2 symmetry, and then high-frequency noise was removed in the Fourier space using a filter at 20 Å.

By using the Euler angles determined (Table I) and the seventeen images two-fold symmetrized, and by using the “make 3D” program in the eman package, a three-dimensional model was reconstructed (Fig. 6a, b). For the same reason as has been described for the conventional EM images, averaging with mirror images was carried out, effectively by further imposing a two-fold symmetry inside each unit, and by creating overall 4·2·2 symmetries.

In the 3D model reconstructed (Fig. 6a, b), four densities (i.e. pairs of N-domains unseparated) were placed with a diameter of ~100 Å on a 2D plane, while the center was left unfilled. Into this center, the crystal structure13),14) of tetrameric assembly of DM1, equivalent to the tetrameric assembly of dimers of C-domains of FL11, was fitted by using the Scene Viewer program25) by coinciding the symmetry axes of the two structures (Fig. 6c, d). Here the isosurface of the FL11 assembly was chosen so that its volume became the same as the Van der Waals volume of the DM1 assembly: the two domains of FL11 have similar molecular weights. Notably DM1 is a variant FFRP, having an assembly domain only (i.e. DM for demi), while the standard FFRP such as FL11 has two domains: the C-domain equivalent with the whole DM1 and an additional N-domain.

The above reconstruction was carried out by using orientations manually identified in real space (Table I). In order to validate this process, automated 3D reconstruction was tried using no manual assignment for orientation but using Fourier correlation only: programs such as StartAny or Startcsym in the eman package were used by assuming a four-fold symmetry. In many models, reconstructed four domains, more or less separated from each other, were arranged on a 2D plane, although details, e.g. the thickness of the model, the presence/absence and the size of the hole in the center, were different (see an example shown in Fig. 6e, f). In one model only two densities were created by selecting part of the images, and in another model, a gyroscope-like structure was made by creating not one but two disks, crossing perpendicular to each other.

## Possible formation of hexameric assemblies

Some particles observed in the electron micrographs were larger than the tetrameric assemblies discussed: the ratio of the two diameters was approximately 1.35. The original (Fig. 3b, upper panels) and two-fold symmetrized (Fig. 3b, lower panels) images of these particles are not inconsistent with the idea of their forming hexameric assemblies. Using the frame in Fig. 5, the Euler angles were determined (Table II). Because of the expected six-fold symmetry, the Δ*ω* parameter now needed to be defined as the average deviation from 60° but not 90°. Observed Δ*ω* was very small, 1.1°–2.5° (Table II), consistent with the idea that the particles projected into these images share a hexagonal symmetry.

The dark densities observed at the centers of the images (Fig. 3b1, 2) might suggest that cavities formed at the centers, sometimes can function as kinds of wells to reserve the dye.

Since FL11 forms a cylinder-like assembly when crystallized, where each six dimers form a helical turn,2) hexamers once formed might further assemble into a cylinder by slightly changing the arrangements of constituent dimers, similarly to another transition observed with tobacco mosaic virus.26)

In the original electron micrographs, a large number of particles much smaller than those discussed in this paper as tetrameric or hexameric assemblies were observed.5) It is known that the structural units of FFRPs are generally dimers,1) and higher-order structures, e.g. particles and cylinders, are assembled by such dimers. The fact that dimers are the predominant assembling form of FL11 has been confirmed by measuring light scattering (Sakuma, M. *et al*., in preparation), and by using gel filtration (Sakuma, M. *et al*., in preparation) and ultra-centrifugation (Arisaka, F. *et al*., in preparation). Estimation of particle sizes using Fourier transform of electron micrographs5) is consistent with the idea that the smallest particles, distributing through the electron micrographs as the “backgrounds”, are images of dimers of FL11.

## Figures and Tables

**Table I:** Parameters for describing projection of the tetrameric assemblies

No	ID	*l*y (l*): *l*x	*θ* x(°)	*θ_ζ_*(°)	Δ*ω*(°)	Figure
T1	b11-12-3-1	148:148	0	89.7	0.3	
T2	c9-12-2	148:148	0	87.8	2.2	3a1
T3	9-12-3-0	140:148	18.9	79.0	1.0	3a2
T4	12-12-1-9	120:148	35.8	89.0	1.2	
T5	10-12-3-5	120:148	35.8	0.0	0.0	
T6	d9-12-1-4	120:148	35.8	89.5	0.5	
T7	11-12-2-10	110:148	42.0	59.0	1.2	3a3
T8	b9-12-3-10	100:148	47.5	53.7	1.7	
T9	11-12-9-2	110:148	42.0	0.8	0.8	3a4
T10	12-12-2-15	110:148	42.0	2.0	0.9	
T11	12-12-3-5	110:148	42.0	81.8	0.1	
T12	11-12-5-4	90:148	52.6	2.5	0.9	
T13	9-12-5-0	80:148	57.3	34.0	0.1	
T14	d9-12-13	70:148	61.8	44.4	1.2	
T15	c12-12-1	60:148	66.1	34.0	1.8	3a5
T16	d9-12-12-2	148*:148	90.0	45.0	N.D.	
T17	d12-12-2	107*:148	90.0	88.8	N.D.	3a6

N.D.: for the two images T16 and T17, *θ* x was set to 90°, and thus the *l*y lengths were closest to 0 and the *ω* angles were not determined (N.D.), so the l* lengths were measured instead by connecting densities along the x axes in order to determine *θ_ζ_* angles.

**Table II:** Parameters for describing projection of the hexameric assembly

No	ID	*l*y (l*): *l*x	*θ* x(°)	*θ_ζ_*(°)	Δ*ω*(°)	Figure
T101	b9-16-e1	180:200	25.8	8.0	2.5	3b1
T102	10-16-6-7	170:200	31.8	23.0	1.1	3b2
T103	b9-16-e3	173*:200	90.0	30.0	N.D.	3b3

N.D.: for image 103, *θ* x was set to 90°, and the *l*y length was closest to 0 and the *ω* angles was not determined (N.D.), so the l* length was measured instead by connecting densities along the x axis in order to determine *θ_ζ_* angle.
